# First report of Anopheles stephensi in Southern Ethiopia

**DOI:** 10.21203/rs.3.rs-3052835/v1

**Published:** 2023-06-15

**Authors:** Dawit Hawaria, Solomon Kibret, Daibin Zhong, Ming-Chieh Lee, Kidane Lelisa, Belayneh Bekele, Muntasha Birhanu, Mathe Mengesha, Hiwot Solomon, Delenesaw Yewhalaw, Guiyun Yan

**Affiliations:** Hawassa University; West Valley Mosquito and Vector Control District; University of California at Irvine; University of California at Irvine; Dilla University; Sidama Regional Health Bureau; Hawassa City Administration Health Department; Sidama Regional Public Health Institute; Ministry of Health; Jimma University; University of California at Irvine

**Keywords:** Anopheles stephensi, malaria, mosquito breeding, mosquito habitat, Ethiopia

## Abstract

**Background:**

*Anopheles stephensi* is an emerging exotic invasive urban vector of malaria in East Africa. The World Health Organization recently announced an initiative to take concerted actions to limit this vector’s expansion by strengthening surveillance and control in invaded and potentially receptive territories in Africa. This study sought to determine the geographic distribution of *An. stephensi* in southern Ethiopia.

**Methods:**

A targeted entomological survey, both larvae and adult, was conducted in Hawassa city, Southern Ethiopia between November 2022 and February 2023. *Anopheles* Larvae were reared to adults for species identification. CDC light traps and BG Pro traps were used overnight both indoor and outdoor at selected houses to collect adult mosquitoes in the study area. Prokopack Aspirator was employed to sample indoor resting mosquitoes in the morning. Adults of *An. stephensi* was identified using morphological keys, and then confirmed by PCR.

**Results:**

Larvae of *An. stephensi* were found in 28 (16.6%) of the 169 potential mosquito breeding sites surveyed. Out of 548 adult female *Anopheles* mosquitoes reared from larvae, 234 (42.7%) were identified to be *An. stephensi* morphologically. A total of 449 female anophelines were caught, of which 53 (12.0%) were *An. stephensi*. Other anopheline species collected in the study area included *An. gambiae* (s.l.), *An. pharoensis*, *An. coustani*, and *An. demeilloni.*

**Conclusion:**

The study, for the first time, confirmed the presence of *An. stephensi* in southern Ethiopia. The presence of both larval and adult stages of this mosquito attest that this species established a sympatric colonization with native vector species such as *An. gambiae* (s.l.) in Southern Ethiopia. The findings warrant further investigation on the ecology, behavior, population genetics, and role of *An. stephensi* in malaria transmission in Ethiopia.

## Background

*Anopheles stephensi* is the principal malaria vector of urban environments in Southeast Asia, the Middle East, and the Arabian Peninsula [1]. Since its initial discovery in Djibouti in 2012 [2], this vector species has been found spreading to eastern Ethiopia [3, 4], Sudan [5], Somalia [1], and Kenya [6].

Its adaptation to urban environment [7] coupled with the rapid spread of *An. stephensi* in Eastern Africa poses serious challenges for the control and elimination of malaria in African’s rapidly urbanizing nations. Malaria outbreak has been documented following its recent colonization of the Horn of Africa region [8] and a 40-fold increase in urban malaria incidence reported in Djibouti over the last decade [9]. According to geospatial modeling studies, many cities in sub-Saharan Africa have environmental conditions favorable for *An. stephensi* to proliferate, putting an additional 126 million Africans at risk of contracting malaria if the spread of *An. stephensi* is not curbed [10]. Another modeling study predicted that if a tailor-made *An. stephensi* interventions are not implemented, the number of malaria cases in Ethiopia might rise by 50% per a year [11].

Determining the geographic range of *An. stephensi* helps design tailored vector interventions. Given the importance of *An. stephensi* to transmit urban malaria and its potential effects on public health in Africa, the World Health Organization (WHO) recently launched an initiative to take coordinated actions to limit the vector’s spread by improving *An. stephensi* surveillance and control in Africa [1]. While the spread of *An. stephensi* has recently been documented in several localities in Eastern, north eastern, and central Ethiopia [3, 4], its occurrence and distribution in Southern Ethiopia is unknown. Thus, in response to WHO call, we conducted an entomological survey to detect and determine the geographic distribution of *An. stephensi* in Hawassa city, Southern Ethiopia.

## Methods

### Study sites

**Figure 1:**
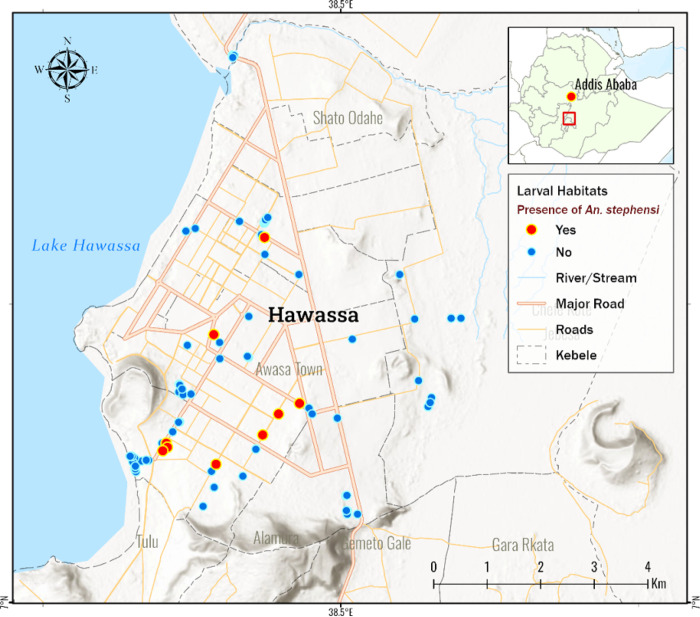
Map of the study site and *Anopheles stephensi* larval habitat distribution

Entomological survey was conducted from November 2022 to February 2023 in Hawassa City in Southern Ethiopia (Fig. 1). Hawassa City is the largest city located in the middle of the Great Ethiopian Rift Valley and is the capita of Sidama Regional State. It is located at the elevation of 1,708 meters (5,604 ft) above sea level. The City’s population was 502,980 in 2022 [12]. Being situated at the shore of Lake Hawassa, the city has seen rapid growth in recent years as it is destined to be one of Ethiopia’s industrial-hub in Southern Ethiopia. The City constantly sees new construction sites following its swift industrial boom in recent years. The average annual temperature and precipitation in the areas is about 21°C and 961 mm, respectively. A shorter rainy season occurs between March and May followed by a longer wet season between July and October. Malaria is seasonal in the area and the main malaria transmission period lies between September and December [12].

### Malaria profile review

Laboratory confirmed malaria data was obtained from Hawassa City Health Department. The malaria morbidity data, which was sorted by age, sex, parasite species and residence for the previous nine years was collected and analyzed, and the annual transmission pattern was established.

### Mosquito larval survey and rearing

Any water collections were surveyed for mosquito larvae and pupae between November 2022 and February 2023. To increase the likelihood of discovering *An. stephensi*, target sampling was conducted. Mosquito larvae and pupae of were dipped from likely larval breeding habitats including man-made water containers, freshwater pools, lake margins, discarded tires, plastic containers and concrete water collection tankers at construction sites. Dipping was done in accordance with larval search strategies recommended by WHO [13]. The larvae and pupae were brought in jars to the environmental lab at Hawassa University, where they were placed in trays and raised to adult in preparation for morphological identification. Each enamel tray containing larvae was labeled by habitat type from where the larvae were obtained in order to identify the species after adult emergence. The larvae were allowed to develop in the water that was drawn from the field in order to maintain the same aquatic environment. The pupae were sorted and transferred with pipettes from the enamel trays to beakers with modest volumes of water, then kept inside cages. A dissecting microscope was used to identify emerging adults to the species using identification key [14]. All identified specimens were preserved individually in Eppendorf tubes for further analysis.

### Adult mosquito survey

Three different types of traps were used to collect adult mosquitoes: CDC Light Traps (Model: John W. Hock CDC Light Trap 512, USA); Bioagents (BG-pro) Traps with lure; and Prokopack Aspirator (John W. Hock 1418, USA).

Collection with CDC Light Trap and BG-pro were made overnight from 18:00 to 06:00 h. A trap was suspended 1.5 meters above the ground in close proximity to a sleeping area where the people are protected by LLINs. Prokopack Aspirator was employed to sample indoor resting mosquitoes in the morning from 6:30 h to 8:00 h in 60 houses.

All collected adult mosquitoes were brought to the lab for species identification. Mosquitoes were killed by placing them in a refrigerator. The specimens were then sorted into culicines and *Anopheles*. Culicine were counted, recorded, and discarded. All anophelines were further sorted out to species using morphological key [14].

### Molecular identification of *An. stephensi*

A subset of the morphologically identified *An. stephensi* specimens were molecularly analyzed in order to confirm the species. DNA was extracted from single leg using the Chelex method [15] with modification. Two methods were used to identify the species: (i) PCR endpoint assay using the internal transcribed spacer 2 (ITS2) locus; and (ii) sequencing portions of cytochrome c oxidase subunit 1 (cox1) and cytochrome B gene (cytb) loci. ITS2 endpoint assay was performed as previously described using the primers 5.8SB (5′-ATG CTT AAA TTT AGG GGG TAG TC-3′) and 28SC (5′-GTC TCG CGA CTG CAA CTG-3′) and the following modifications: final reagent concentrations and components were 0.5 μM for each primer; 1× DreamTaq Green Master Mix (Thermo Fisher Scientific); and water for a total reaction volume of 17 μl. PCR reaction conditions were set as denaturation at 95 °C for 3 min, 35 cycles of 94 °C for 30 s, annealing at 55 °C for 30 s, extension at 72 °C for 30 s, and a final step at 72 °C for 6 min. *Anopheles stephensi* specimens were identified by visualization of 522-bp band with gel electrophoresis; non-*An. stephensi* specimens do not amplify and no band was present [16]. Portions of the cox1 and cytb loci were also amplified for sequencing using previously detailed methods [17]. PCR products were purified and sequenced using Sanger technology by Genewiz Inc (South Plainfield, NJ). Sequences were cleaned and analyzed using CodonCode (CodonCode Corporation, Centerville, MA, USA). Next, cox1 and cytb sequences from *An. stephensi* were submitted as queries to the National Center for Biotechnology Information’s (NCBI) Basic Local Alignment Search Tool (BLAST) [18] against the nucleotide collection in NCBI’s GenBank. A threshold limit of 98% sequence similarity for cox1 was used to classify sequences into species [19].

## Results

### Malaria morbidity

**Figure 2:**
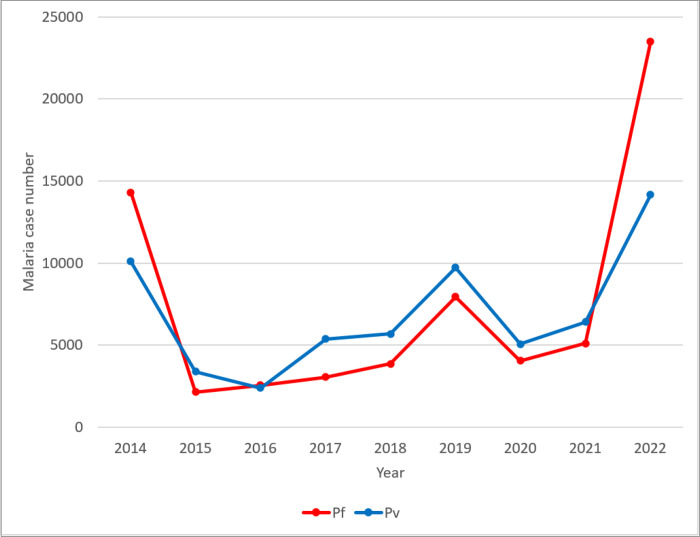
Annual malaria cases in Hawassa City, Southern Ethiopia, 2023

Over the last nine years, 128,946 malaria cases were reported in the study area. *Plasmodium falciparum* and *P. vivax* were prevalent in the area and nearly had identical proportions with 66,570 (51.6%) and 62,376 (48.4%), respectively. According to the data, malaria transmission was dropping for three years in a row, from 2014 to 2016. However, from 2017 to 2019, there was slight increment. Then, after a drop in year 2020, number of malaria cases skyrocketed in 2022 (Fig. 2).

### Larval habitat distribution, positivity, and *Anopheles* species composition

*Anopheles stephensi* larvae were detected in 28 (16.6%) of the 169 mosquito breeding sites surveyed. The frequent habitats where *An. stephensi* was discovered were water tanks, including plastic and metal, concrete water cisterns at construction sites, and car wash facilities (Table 1). During the survey, 2,012 Anopheles larvae were collected and reared, and 548 (27.7%) adult females *Anopheles* were emerged. Morphologically, 234 (42.7%) of 558 were identified to be *An. stephensi*. Other *Anopheles* species identified included, *Anopheles gambaie* (s.l.) (n=167; 30.5%), *An. Pharoensis* (n=98; 17.9%), and *An. counstani* (n=49; 8.9%). Fig. 1 depicts the distribution of *An. stephensi*’s breeding habitats.

### Adult collection’s *Anopheles* species composition

**Figure 3:**
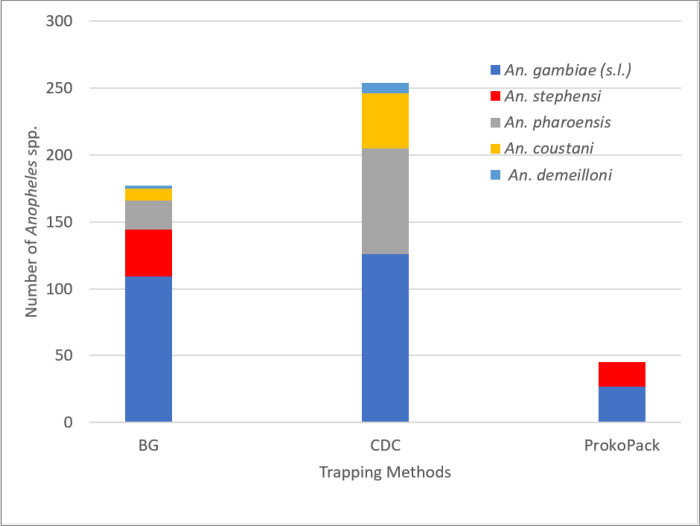
Number of *Anopheles*species caught in different trapping methods, Hawassa City, Southern Ethiopia, 2023

The adult survey captured 449 female *Anopheles*, 53 (12.0%) of them were *An. stephensi*. *Anopheles stephensi* was captured by the BG Pro Trap and the ProkoPack Aspirator. No *An. stephensi* was captured by the CDC light trap. *Anopheles gambiae* (s.l.), *An. pharoensis, An. coustani*, and *An. demeilloni* were other *Anopheles* species collected during the survey. *Anopheles gambiae* (s.l.) was the predominant species (53.7%) followed by *An. pharoensis* (23.3%) and *An. coustani* (11.5%) (Fig. 3).

### Molecular identification of *An. stephensi*

Of the 50 morphologically identified *An. stephensi* specimens analyzed, ITS2 PCR results were obtained from all of them. We compared the morphological identification to the ITS2 PCR endpoint assay results. With the PCR endpoint assay, 48 (96%) specimens were confirmed as *An. stephensi*. Only two of the 50 (4.0%) morphologically identified *An. stephensi* were not confirmed with the PCR endpoint assay. Sanger sequencing was performed for 30 confirmed and 2 not confirmed specimens. Of the 30 *An. stephensi*, 11 were found to be cox1 haplotype 2 (GenBank accession OQ865406) and cytb haplotype 2 (GenBank accession OQ863377), while 19 were cox1 haplotype 3 (GenBank accession OQ865407) and cytb haplotype 1 (GenBank accession OQ863376). The two specimens not confirmed by ITS2 PCR were identified as *An. arabiensis* by cox1 sequencing.

## Discussion

The recent colonization of *An. stephensi* in Eastern Africa has called for additional molecular surveillance to delineate the expansion of this new species in Africa [3,4]. To the best of our knowledge, this is the first report to confirm the presence of *Anopheles stephensi* in Southern Ethiopia. Recent increases in malaria transmission in this region coincided with the expansion of this species in Ethiopia.

Such rapid spread of *An. stephensi* to many areas of Ethiopia triggers the question of whether it is truly new arrival or whether it has been present for a while but gone unnoticed. Several hypotheses have been forwarded by the scholars, one of which is that *An. stephensi* was recently brought to Ethiopia from neighboring country Djibouti which Ethiopia uses as a major good importation route [10]. According to the present data, the Ethiopian isolate is most closely related to an isolate from Pakistan [4]. The second hypothesis involved *An. stephensi* being present in Ethiopia for a long period yet gone unnoticed. Provided the existing gaps in molecular vector surveillance, it is necessary to carefully re-examine the trend of entomological surveillance practices that entirely base on morphological keys for species identification. The morphological similarity of *An. stephensi* to other Anopheles species, such as *An. arabiensis* as documented in this study, plus the infrequent utilization and cost of molecular methods for identification, such as PCR and sequencing, could possibly explain why the species have been overlooked [4,20]. This study was carried out in Southern Ethiopia, which is far from the location where *An. stephensi* colonization was initially recorded in the country [4], suggesting that *An. stephensi* colonization is still ongoing. This finding underscores the critical need to expand molecular vector surveillance in all malaria-prone areas in order to map *An. stephensi*’s geographic distribution in the country.

Water tanks at construction site, concrete water cisterns, and concrete water collection boxes for carwashes were the frequent habitats found to harbor *An. stephensi* in the study area. All these breeding sites were man-made, produced as a result of urbanization. Hawassa is Ethiopia’s major cities, destined to be an industrial zone and rapidly expanding in recent years. Massive development projects are currently undergone, opening the door for the proliferation of new *An. stephensi* breeding environments. Building strong collaborations across many sectors, including as health, trade and industry, housing agencies, education, and municipalities, is critical to achieve successful vector control responses in such metropolitan contexts. Previous studies in several countries reported that artificial containers are an appropriate breeding site for *An. stephensi* [3,4,21,22]. As the World Health Organization recommends, an integrated vector control strategy should be promoted in the area [1].

Discovering *An. stephensi* in Hawassa has important public health implications. A coincidental observation during the surveillance was the city’s rising malaria cases after a decade-long decline. *Plasmodium vivax* and *P. falciparum* are both actively transmitted in the study area. Due to its vectorial capacity to spread both *P. falciparum* and *P. vivax* [7], the spread of *An. stephensi* might been contributing to increasing malaria transmission in the Southern Ethiopia. Studies have indicated that malaria incidence increased following the establishment of *An. stephensi* in eastern Ethiopia and Djibouti [8,9]. Further research is required to determine the relative contribution of *An. stephensi* to the city’s rising malaria incidence because it coexists alongside native vector species such as *An. arabiensis*, *An. coustani*, and *An. pharoensis.* The survey result suggests the need for further study to elucidate the ecology, behavior, and population genetics of this invasive species. Better understanding on the extent of its distribution and its role in malaria transmission helps develop tailor-made vector intervention strategies in urban settings.

## Conclusion

*Anopheles stephensi* coexists with native vector species in Southern Ethiopia. Further research is needed to determine its relative role in malaria transmission in the region. Heath authorities need to revise the existing vector control strategies to target *Anopheles stephensi* alongside the native malaria vector species.

## Figures and Tables

**Table 1:** *Anopheles* mosquito larval habitat positivity and species composition by habitat types, Hawassa, southern Ethiopia, 2023

Habitat types	Surveyed habitats n (%)	Positive for any mosquito larvae n (%)	Positive for *Anopheles* Larvae n (%)	Positive for *An. stephensi* n (%)	*Anopheles* spp.
Water tanks	57 (31.5)	24 (42.1)	20 (35.1)	20 (35.1)	• *An. stephensi*
Concrete water cisterns at construction sites	44 (26.2)	20 (45.5)	11 (25.0)	6 (13.6)	• *An. stephensi and An. gambiae (s.l.)*
Concrete water collection box for carwash	10 (6.0)	3 (30.0)	2 (20.0)	2 (20.0)	• *An. stephensi*
Shoreline / lakeadge	9 (5.4)	8 (88.9)	7 (77.7)	no	• *An. gambiae (s.l.), An. pharoensis, and An. coustani*
Drainage ditch	8 (4.8)	4 (50.0)	2 (25.0)	no	• An. gambiae (s.l.)
Discarded buckets	7 (4.2)	3 (42.8)	2 (28.6)	no	• An. gambiae (s.l.)
Manmade pools	5(3.0)	no		no	
Swamps/marshes	4 (2.4)	4 (100.0)	4 (100.0)	no	• *An. gambiae (s.l.), An. pharoensis, and An. coustani*
Discarded tires	4 (2.4)	2 (50.0)	no	no	
Tire tracks/Road paddles	4 (2.4)	2 (50.0)	2 (50.0)	no	• *An. gambiae (s.l.)*
Excavated ground for road construction	3 (1.8)	3 (100.0)	3 (100.0)	no	• *An. gambiae (s.l.)*
Others	14 (8.3)	7 (50.0)	1 (7.1)	no	

Abbreviation: no, not observed; spp., species

## Data Availability

The datasets used and/or analyzed during the current study are available from the corresponding author on reasonable request.

## References

[1] World Health Organization. WHO initiative to stop the spread of Anopheles stephensi in Africa. 2022;4. Available from: https://www.who.int/publications/i/item/WHO-UCN-GMP-2022.06

[2] Faulde MK, Rueda LM, Khaireh B. First record of the Asian malaria vector Anopheles stephensi and its possible role in the resurgence of malaria in Djibouti, Horn of Africa. Acta Trop. 2014;139:39–43. 25004439 10.1016/j.actatropica.2014.06.016

[3] Balkew M, Mumba P, Dengela D, Yohannes G, Getachew D, Yared S, Chibsa S, Geographical distribution of Anopheles stephensi in eastern Ethiopia. Parasit Vectors. 2020;13:1–8. 31959237 10.1186/s13071-020-3904-y PMC6971998

[4] Carter TE, Yared S, Gebresilassie A, Bonnell V, Damodaran L, Lopez K, First detection of Anopheles stephensi Liston, 1901 (Diptera: culicidae) in Ethiopia using molecular and morphological approaches. Acta Trop. 2018;188:180–6. 30189199 10.1016/j.actatropica.2018.09.001

[5] Ahmed A, Pignatelli P, Elaagip A Invasive Malaria Vector *Anopheles stephensi* Mosquitoes in Sudan, 2016–2018. Emerg Infect Dis. 27:2952–4. 10.3201/eid2711.210040 PMC8544976 34670658

[6] Ochomo Eric O., Sylvia Milanoi BA Molecular surveillance leads to the first detection of Anopheles stephensi in Kenya, 20 January 2023, PREPRINT (Version 1) available at Research Square [10.21203/rs.3.rs-2498485/v1].

[7] Tadesse FG, Ashine T, Teka H, Esayas E, Messenger LA, Chali W, Anopheles stephensi Mosquitoes as Vectors of Plasmodium vivax and falciparum, Horn of Africa, 2019. Emerg Infect Dis. 2021;27:603–7. 33496217 10.3201/eid2702.200019 PMC7853561

[8] Vogel G. Invasive mosquito adds to Africa’s malaria toll. Science. 2022;378:582–3. 36356129 10.1126/science.adf7188

[9] de Santi VP, Khaireh BA, Chiniard T Role of *Anopheles stephensi* Mosquitoes in Malaria Outbreak, Djibouti, 2019. Emerg Infect Dis. 2021;27:1697–700. 34013869 10.3201/eid2706.204557 PMC8153885

[10] Sinka ME, Pironon S, Massey NC, Longbottom J, Hemingway J, Moyes CL, A new malaria vector in Africa: Predicting the expansion range of *Anopheles stephensi* and identifying the urban populations at risk. Proc Natl Acad Sci U S A. 2020;117:24900–8. 32929020 10.1073/pnas.2003976117 PMC7547157

[11] Hamlet A, Dengela D, Tongren JE, Tadesse FG, Bousema T, Sinka M, The potential impact of Anopheles stephensi establishment on the transmission of Plasmodium falciparum in Ethiopia and prospective control measures. BMC Med. 2022;20:1–10. 35440085 10.1186/s12916-022-02324-1 PMC9020030

[12] Hawassa City Heath Department. Annual Malaria Morbidity report. Hawassa; 2022.

[13] WHO: Manual on practical entomology: part II method and techniques. Geneva: World Health Organization; 1975.

[14] Coetzee M. Key to the females of Afrotropical Anopheles mosquitoes (Diptera: Culicidae). Malar J. 2020;19:1–20. 32054502 10.1186/s12936-020-3144-9 PMC7020601

[15] Onyango SA, Ochwedo KO, Machani MG, Olumeh JO, Debrah I, Omondi CJ, Molecular characterization and genotype distribution of thioester-containing protein 1 gene in *Anopheles gambiae* mosquitoes in western Kenya. Malar J. 2022;21:1–10. 35948910 10.1186/s12936-022-04256-w PMC9364548

[16] Balkew M, Mumba P, Dengela D, Yohannes G, Getachew D, Yared S, Geographical distribution of Anopheles stephensi in eastern Ethiopia. Parasites and Vectors. 2020;13:1–8. 31959237 10.1186/s13071-020-3904-y PMC6971998

[17] Carter TE, Yared S, Hansel S, Lopez K, Janies D. Sequence-based identification of Anopheles species in eastern Ethiopia. Malar J. 2019;18:135. 30992003 10.1186/s12936-019-2768-0 PMC6469081

[18] Altschul SF, Gish W, Miller W, Myers EW LD. Basic local alignment search tool. J Mol Biol. 1990;215:403–10. 2231712 10.1016/S0022-2836(05)80360-2

[19] Lobo NF, Laurent B, Sikaala CH, Hamainza B, Chanda J, Chinula D, Unexpected diversity of Anopheles species in Eastern Zambia: Implications for evaluating vector behavior and interventions using molecular tools. Sc Rep. 2015;5:1–10. 10.1038/srep17952 PMC4673690 26648001

[20] Mnzava A, Monroe AC, Okumu F. Anopheles stephensi in Africa requires a more integrated response. Malar J. 2022;21:4–9. 35641958 10.1186/s12936-022-04197-4 PMC9152833

[21] Gayan Dharmasiri AG, Perera AY, Harishchandra J, Herath H, Aravindan K, Jayasooriya HTR, First record of Anopheles stephensi in Sri Lanka: a potential challenge for prevention of malaria reintroduction. Malar J. 2017;16:326. 28797253 10.1186/s12936-017-1977-7 PMC5553605

[22] Manouchehri A V., Javadian E, Eshighy N, Motabar M. Ecology of *Anopheles stephensi* Liston in southern Iran. Trop Geogr Med. 1976;28:228–32. 1006792

